# The Role of the Summit in Global Health Governance: A Neglected Way

**Published:** 2020-02

**Authors:** Wenwen WU, Xiaodong TAN, Yu XIE, Qiru WANG, Yalin ZHANG

**Affiliations:** Department of Occupational and Environmental Health, School of Health Sciences, Wuhan University, Wuhan, China

## Dear Editor-in-Chief

The economic globalization, characterized by free movement of goods, capital, technology, labor and information, is becoming more and more universal in the 21st century, making the whole world a “global village” (1, 2). The effect of globalization on public health problems is more presented. It makes it possible for certain health events in one country or region to rapidly affect other countries and regions. The health and security of the entire international community has become an integral whole (3). In response to the global outbreak of major infectious diseases in the early 21st century, global health governance has attracted international attention (4). “The Constitution of the World Health Organization (1946)” states that “the enjoyment of the highest level of health is the fundamental right of everyone and is not different from race, religion, political belief, economic or social context”(5). Global health governance is based on the right of health, and its mission is to promote the global realization of “health for all” by curbing the crisis that globalization poses to human health.

### An analysis of the themes of summits in 2016

**Fig. 1:**
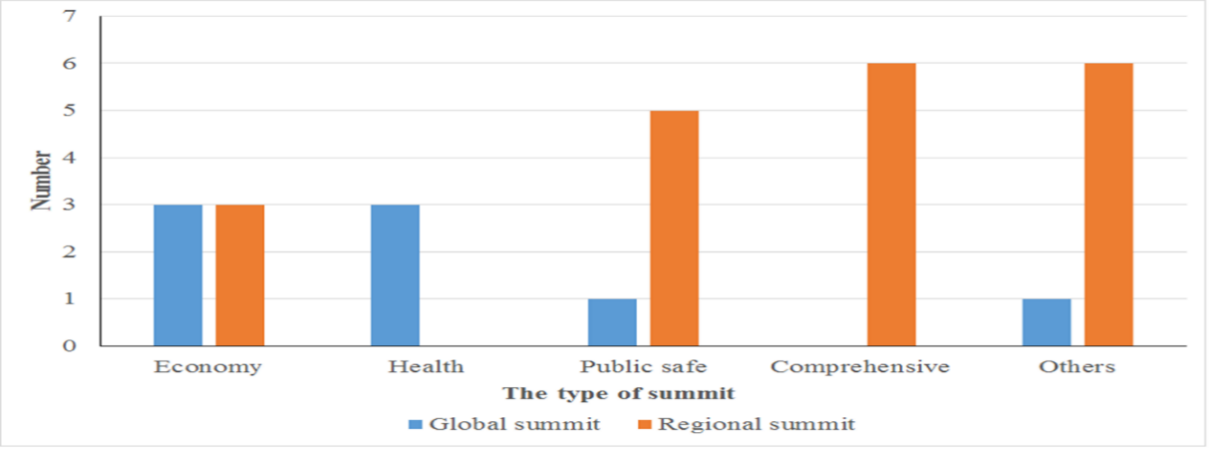
Summits held in 2016

Different types of summits are important platforms for promoting global health governance. In a narrow sense, the summit refers to a leadership conference between a country or an international organization, usually including global summits and regional summits (6). The globalization of health makes global health governance inevitable, and the G8, G20, BRICS, the EU have already involved the health into the part of UN Summit Diplomacy. Figure 1 shows that there were 28 summits were held around the world concerned different themes in 2016.

**Fig. 2:**
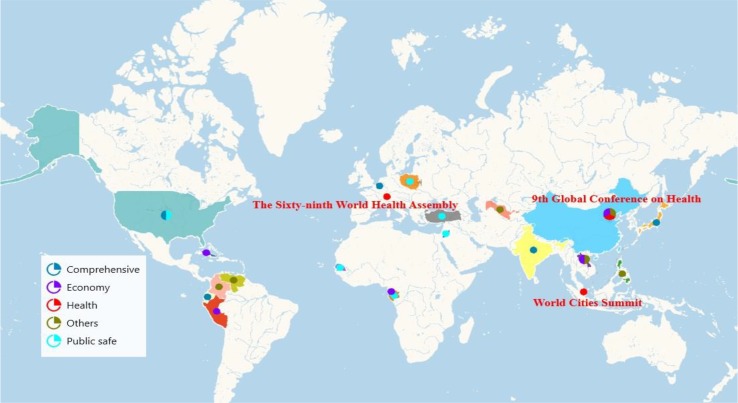
Health-related summit geographical distribution

Classified by subject, there were 9 globally while 19 regionally. Among these summits, 6 were economic related, 6 were public safe related, 6 were comprehensive assembly, and 7 were others assembly. However, there were only 3 of them held by Global International Organization whose theme were health (Fig. 2).

### Why are there so few health-themed summits?

While health is a key area of constant concern, there is a serious lack of opportunity to discuss this topic on an important international diplomatic platform. The reasons accounting for the results are complicated. Firstly, the authoritative role of global health governance has not yet been formed, without a strong summit organizer. Secondly, the diversity of health problems leads to different concerns about different health issues, and the willingness of countries to participate in conferences is difficult to obtain consistency. In such situation, it is difficult for a conference to have the scale or level or influence it should have as the summit. Thirdly, in recent years, terrorism, refugee crisis, economic and financial crisis and other issues that affecting national stability and security, have became the preferred cooperation issues.

### The functions of the summit in global health governance

However, this cannot manifest that the global health governance is meaningless and worthless. Hosting health-themed summits would have at least three functions for global health governance. Firstly, the summit can gather strength and make it easier to solve health problems that require cooperation between countries. Secondly, the summit can contribute to the formulation of global health governance standards and health technical norms. Moreover, based on the consideration of the cultural, ethnic, religious values and policies of different countries, summits can promote research on global health issues in the biomedical and health services system, and expand the application of research results. In addition, summits can facilitate the worldwide information share and some countries can learn from the lessons of other countries.

### How to make better use of summit in global health governance

With the multi-polarization of global politics, the world also needs to actively establish the core leaders of global or regional health governance (7) and act as the organizer of the summit. Both developed and developing countries need to view from the benefit of long term, adapt to the global trend of health governance, and actively advocate healthy themed summits. We also need to promote diversity in the theme of the global health governance summit and form a long-term mechanism for regular summits. In addition, health should be considered as one of the themes to be included as much as possible in other global or regional summits.
